# Eradication of *Burkholderia cepacia* Using Inhaled Aztreonam Lysine in Two Patients with Bronchiectasis

**DOI:** 10.1155/2014/192146

**Published:** 2014-09-09

**Authors:** A. Iglesias, I. Artiles, J. J. Cabanillas, R. Álvarez-Sala, C. Prados

**Affiliations:** Cystic Fibrosis-Bronchiectasis Unit, Pulmonology Service, La Paz University Hospital, 28046 Madrid, Spain

## Abstract

There are not many articles about the chronic bronchial infection/colonization in patients with underlying lung disease other than cystic fibrosis (CF), especially with non-CF bronchiectasis (NCFBQ). The prevalence of *B. cepacia complex* is not well known in NCFBQ. The vast majority of published clinical data on *Burkholderia* infection in individuals with CF is comprised of uncontrolled, anecdotal, and/or single center experiences, and no consensus has emerged regarding treatment. We present two cases diagnosed with bronchiectasis (BQ) of different etiology, with early pulmonary infection by *B. cepacia complex*, which was eradicated with inhaled aztreonam lysine.

## 1. Introduction

The* Burkholderia cepacia complex *is a group of 17 closely related bacterial species, most of them causing life-threatening infections in patients with CF, granulomatous disease, and immunodeficiencies and those undergoing mechanical ventilation [1, 2]. However, there are not many articles about the chronic bronchial infection/colonization in patients with underlying lung disease other than CF, especially with non-CF bronchiectasis (NCFBQ). The prevalence of* B. cepacia complex* is not well known in NCFBQ [1]. Currently, we have not found any recommendations for the treatment of* B. cepacia complex*. The vast majority of published clinical data on* Burkholderia* infection in individuals with CF is comprised of uncontrolled, anecdotal, and/or single center experiences, and no consensus has emerged regarding treatment [3]. Some authors have found that the use of a combination of nebulised tobramycin and amiloride could suppress or potentially eradicate* B. cepacia complex*, at least in some instances [4]. Currently, the data from the clinical trial about the CF patients with chronic* B. cepacia complex* infection treated with inhaled aztreonam lysine continuously were published without obtaining good results [5].

We present two cases diagnosed with BQ of different etiology, with early pulmonary infection by* B. cepacia complex*, which was eradicated with inhaled aztreonam lysine. The future of nebulised antibiotic therapy, especially of this antibiotic, not only for the pulmonary infection by* P. aeruginosa* but also for other respiratory infections in bronchiectasis, may be very promising.

## 2. Case Reports

### 2.1. Case 1

The case is a 77-year-old man whose personal history included arterial hypertension, hypercholesterolemia, chronic ischemic cardiopathy, chronic obstructive respiratory disease, bronchiectasis due to IgG2 deficiency, and old pulmonary tuberculosis which was treated by bilobectomy (medial and lower right lobe). He presented a chronic colonization by* P. aeruginosa* treated by inhaled sodium colistimethate since 2006. He attended the clinic for an ordinary revision, and an early bronchial infection by* B. cepacia complex* resistant to colistin and aminoglycosides was discovered. It was treated with inhaled aztreonam lysine, 75 mg, three times daily in a period of 28 days on-off, according to the antibiogram. The microbiological sputum study after 28 days of treatment just detected saprophytic flora, findings that have remained one year after the beginning of the treatment. After the third cycle with inhaled aztreonam and according to the sputum cultures, the antibiotic was suspended.

### 2.2. Case 2

The case is a 26-year-old women diagnosed with CF at the age of 2 years, with a wild-moderate pulmonary disease and a chronic bronchial infection by* P. aeruginosa* treated with inhaled sodium colistimethate, since she was 18 years old. In a visit to the clinic,* B. cepacia multivorans* was discovered in the sputum culture, with resistances to colistin and tobramycin, without any respiratory symptoms. She started inhaled aztreonam lysine 75 mg three times daily in periods of 28 days on-off and inhaled sodium colistimethate during the off period, according to the antibiogram (although it was resistant to colistin). After the first cycle of aztreonam lysine,* B. cepacia multivorans* did not return to be detected in the sputum culture, one year and a half later.

## 3. Discussion

Members of the* Burkholderia cepacia *complex are important pathogens in CF lung disease, infecting about 3% of CF patients worldwide, although it widely varies from center to center. However, it is unclear how prevalent* B. cepacia complex* is in patients with underlying lung disease other than CF. Its importance is associated with significant morbidity and mortality [3, 6–9].* B. cepacia* is often resistant to many antibiotics, displaying both intrinsic and inducible resistance, so antibiotic therapy is generally suppressive, rather than curative [3].

The use of inhaled antibiotics in patients with NCFBQ chronically infected by* P. aeruginosa* is an increasingly common practice, totally indicated, both in the early onset infection and chronic colonization/bronchial infection by* P. aeruginosa*, in CF [10–12]. After the use of inhaled antibiotics in CF, there are a lot of studies whose conclusions indicate that inhaled antibiotics may provide an effective suppressive therapy with an acceptable safety profile in adult patients with stable NCFBQ and chronic bronchial infection [13].

The vast majority of published clinical data on* Burkholderia cepacia complex *infection in individuals with CF is comprised of uncontrolled, anecdotal, and/or single center experiences, and no consensus has emerged regarding treatment. One of the reasons is that individuals with* Burkholderia cepacia complex* infection have historically been excluded from efficacy trials of inhaled antibiotics [5, 8, 9].

Aztreonam lysine (Cayston, Gilead Sciences Inc.) is a new antibiotic for inhalation, indicated for the treatment of chronic bronchial infection by* P. aeruginosa* in CF patients [12, 14]. We have used this antibiotic for the treatment of early bronchial infection by* B. cepacia complex* in patients with BQ due to different etiologies, with very good results, in spite of the results of the clinical trial [5].

For this reason, we think that, due to the limited experience that we have with this antibiotic and this microorganism, these cases could help in the treatment of patients with similar characteristics. More studies are needed to extend the indication of this antibiotic for the treatment of early bronchial infection by other Gram-negative microorganisms, as* B. cepacia complex*, which would be of enormous help to control the bronchial infection by this pathogen, with the aim of a reduction of the morbidity and mortality of patients with BQ due to different etiologies.
